# Mutational profiles associated with resistance in patients with BRAFV600E mutant colorectal cancer treated with cetuximab and encorafenib +/− binimetinib or alpelisib

**DOI:** 10.1038/s41416-020-01147-2

**Published:** 2020-11-18

**Authors:** Sanne C. F. A. Huijberts, Mirjam C. Boelens, Rene Bernards, Frans L. Opdam

**Affiliations:** 1 Department of Clinical Pharmacology, The Netherlands Cancer Institute, Amsterdam, The Netherlands; 2 Department of Pathology, The Netherlands Cancer Institute, Amsterdam, The Netherlands; 3 Department of Molecular Carcinogenesis, The Netherlands Cancer Institute, Amsterdam, The Netherlands; 4 Department of Medical Oncology, The Netherlands Cancer Institute, Amsterdam, The Netherlands

**Keywords:** Colon cancer, Tumour biomarkers

## Abstract

**Background:**

Treatment strategies inhibiting BRAF in combination with EGFR have been developed in patients with *BRAF*^*V600E*^ mutant metastatic colorectal cancer, but intrinsic and secondary resistance remains a challenge. We aimed to investigate which genetic alterations cause intrinsic non-response and/or acquired resistance in these patients receiving therapies consisting of a backbone of BRAF and EGFR inhibition.

**Methods:**

This was a cohort study on genetic alterations in patients with *BRAF*^*V600E*^ mutant advanced colorectal cancer treated with inhibitors of the MAPK pathway. We examined tumour tissue for genetic alterations at baseline, during treatment and at progression.

**Results:**

In total, 37 patients were included in this cohort. Genetic alterations in *EGFR* and in *PIK3CA* are associated with non-response. A greater fraction of non-responders (75%) versus responders (46%) had at least one genetic alteration in other genes than *TP53*, *APC* or *BRAF*. Secondary resistance mutations (*n* = 16 patients) were observed most frequently in the PI3K pathway (*n* = 6) and in receptor tyrosine kinases (*n* = 4), leading to increased upstream signalling.

**Conclusions:**

Genetic alterations in the PI3K and upstream receptor tyrosine kinases were mostly associated with intrinsic and acquired resistance. By understanding these alterations, simultaneous or alternating treatments with targeted inhibitors might improve response duration.

## Background

Colorectal cancer (CRC) is one of the leading causes of mortality in the world and was responsible for almost 900,000 deaths worldwide in 2018.^1^ At initial diagnosis, metastasised disease is found in 25% of the patients and 50% of all patients will develop metastases during their disease course.^2^ Approximately 8–15% of metastatic CRC harbour a *BRAF*^*V600E*^ mutation, which results in failure of standard chemotherapy and a dismal prognosis.^3^ The *BRAF* gene encodes a serine/threonine protein kinase, which is part of the signal transduction pathway RAS-RAF-MEK-ERK, also known as the mitogen-activated protein kinase (MAPK) pathway. Activating *BRAF* mutations are leading to signalling via this pathway by phosphorylation of the downstream MEK 1/2 proteins. MEK 1/2 subsequently phosphorylates the ERK1/2 kinases, resulting in gene transcription that drives cell proliferation and survival.^4^ The *BRAF*^*V600E*^ mutation is the most common mutation among different tumour types and is caused by the substitution of valine to glutamic acid within codon 600.^5^ *BRAF*^*V600E*^ mutations were initially reported by Davies et al. in 2002. They discovered that these mutations in melanoma led to an overactive MAPK pathway and could therefore be an interesting drug target.^6^ During the past decades, researchers intensively studied the *BRAF*^*V600E*^ mutation to understand its role in tumour development and to explore possible treatment strategies for *BRAF*^*V600E*^ mutated carcinoma, including CRC. Initially, the BRAF inhibitor vemurafenib had been investigated with observed responses in only 5% of the patients with *BRAF*^*V600E*^ mutated metastatic CRC.^7^ This lack of response was found to be the result of feedback reactivation of EGFR after BRAF inhibition, thereby limiting the response to BRAF inhibitors.^8,9^ To optimise response rates, BRAF inhibitors have been combined with EGFR inhibitors and other targeted agents in doublet and triplet regimens.^10–13^ So far the combination of the BRAF inhibitor encorafenib and the EGFR inhibitor cetuximab with or without the MEK inhibitor binimetinib showed the best outcome with an overall response rate of 23% (doublet) and 29% (triplet) with manageable toxicity. Progression free survival was 4.2 months for the doublet and 4.3 months for the triplet regimen.^11^ The combination of encorafenib and cetuximab was recently approved by the Food and Drug Administration (FDA).^14^ Although response rates are improved and acquired resistance delayed, progression free survival remains short and resistance is still a major challenge.^15^

Since not all *BRAF*^*V600E*^ mutant tumours are responsive to MAPK inhibitors and resistance patterns differ among preclinical studies, it is likely that *BRAF*^*V600E*^ mutated tumours are highly heterogeneous.^16^ Moreover, two gene expression subtypes were earlier identified in a clustered analysis of 218 biopsies from *BRAF*^*V600E*^ mutant tumours. *BRAF*^*V600E*^ mutant subtype 1 (BM1) harboured KRAS/mTOR/AKT/4EBP1 activation with high levels of immune infiltration and epithelial-mesenchymal transition (EMT) and *BRAF*^*V600E*^ mutant subtype 2 (BM2) was mainly dysregulated in cell-cycle checkpoints.^17^ A higher sensitivity for BRAF, MEK and EGFR inhibition with dabrafenib, trametinib and panitumumab was found in BM1.^18^ These results suggest that *BRAF*^*V600E*^ mutated CRC is indeed a heterogeneous disease with different molecular patterns, responses to and targets for therapy.

Improvement in understanding this heterogeneity, resistance and moderate response rates of MAPK inhibitors in *BRAF*^*V600E*^ mutated CRC is pivotal to optimise treatment outcomes. We here provide an overview of intrinsic and acquired mutations before and during treatment with targeted agents in patients with *BRAF*^*V600E*^ mutant CRC. We present a cohort study of *BRAF*^*V600E*^ mutant CRC patients treated with combinations of MAPK inhibitors in the Netherlands Cancer Institute-Antoni van Leeuwenhoek hospital (NKI-AVL). The research questions we aim to address are; (1) Which biomarkers are associated with non-responders? (2) Which secondary mutations causing acquired resistance are developed during targeted treatment of the MAPK pathway? (3) Can we take advantage of this secondary mutations for optimisation of subsequent treatment?

## Methods

### Cohort study

This retrospective cohort study was conducted in the NKI-AVL in patients with *BRAF*^*V600E*^ mutated metastatic CRC between January 2012 and December 2019. All patients gave permission for the use of their residual material and data for research purposes. All patients included in the analyses were treated with targeted therapies, including the following combinations; encorafenib/cetuximab, encorafenib/cetuximab/alpelisib or encorafenib/cetuximab/ binimetinib. Encorafenib is an orally administered small molecule that inhibits BRAF.^19^ Cetuximab is an intravenously administered monoclonal antibody that binds specifically to the extracellular domain of EGFR. Binimetinib is an orally available small molecule and inhibits MEK 1/2.^20^ Alpelisib is an orally administered small molecule that inhibits PI3-kinase.^10^ The drugs were administered in the following doses: encorafenib 100–450 mg once daily continuously, cetuximab in an initial dose of 400 mg/m^2^ and thereafter 250 mg/m^2^ weekly, binimetinib 45 mg twice daily continuously and alpelisib 100–300 mg once daily continuously. We reviewed electronical medical records for information on demographics, anti-tumour response according to RECIST v 1.1. and mutational status. Results from histopathological reports of tumour biopsies before, during treatment or at progressive disease were collected, if available. On treatment tumour biopsies were collected after at least two weeks of treatment, which means that all drugs were on steady state concentrations. Results from different sequencing methods were included (Supplementary Table S1). If paired biopsies were available for patients, we carefully reviewed the sequencing methods used for overlap in genes present in the panels of the different sequencing methods. All results presented are restricted to genetic alterations of known clinical significance. This means that variants of unknown clinical significance (VUS) were excluded from this analysis.

### Statistical methods

Descriptive statistics, including median along with percentages and frequencies for categorical variables were tabulated and presented in this paper. Responses were defined according to RECIST version 1.1. criteria.^21^ Time to progression was defined as the time from start of targeted treatment to date of first mentioning of progressive disease.

## Results

### Overall baseline characteristics

A total of 53 patients with *BRAF*^*V600E*^ mutated metastatic CRC were screened for mutational status at baseline, on treatment and at progressive disease. The genetic alterations, including the variant of the mutation (e.g. *KRAS*^*G12V*^), anti-tumour response and sequencing method per patient are summarised in Supplementary Table S2 for the total set of 53 patients. The current analysis is performed in 37 patients with at least one other detected genetic alteration at one-time point, besides the known *BRAF*^*V600E*^ mutation.

The majority of patients was pre-treated with no more than two lines of anti-cancer therapy for advanced disease, including one patient with one line of immunotherapy, before the start of double or triple combined targeted agents. Seventeen patients were treated with encorafenib and cetuximab, seven patients with the combination of encorafenib, cetuximab and binimetinib and 13 patients with encorafenib, cetuximab and alpelisib. More than half of the tumours were microsatellite stable (Table 1).

**Table 1:** Baseline characteristics of patients included in the analyses.

	Patients *n* = 37
Sex, *n* (%)	
Female	24 (65%)
Male	13 (35%)
Age, median (range), years	59 (38–74)
Number of prior treatment lines, *n* (%)	
1	14 (38%)
2	17 (46%)
≥3	6 (16%)
Treatment arm, *n* (%)	
Encorafenib + cetuximab	17 (46%)
Encorafenib + cetuximab + binimetinib	7 (19%)
Encorafenib + cetuximab + alpelisib	13 (35%)
Micro satellite stability, *n* (%)	
MSI	1 (3%)
MSS	20 (54%)
Unknown	16 (43%)
Time points molecular analysis, *n* (%)	
Single: BL	16 (43%)
Single: OT	2 (5%)
Paired: BL and OT	5 (14%)
Paired: BL and PD	6 (16%)
Paired: OT and PD	4 (11%)
Paired: BL, OT and PD	4 (11%)

*n* number, *MSI* micro satellite instable, *MSS* micro satellite stable, *BL* baseline, *OT* on treatment, *PD* progressive disease.

**Fig. 1:**
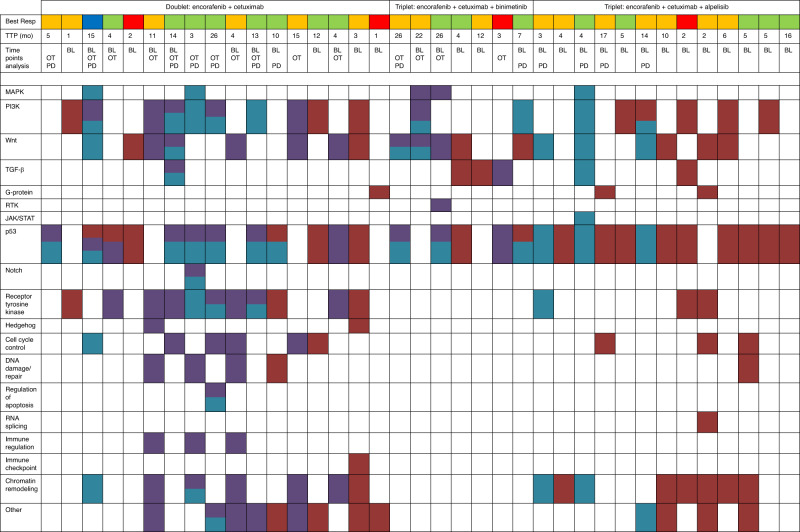
Anti-tumour activity, including best response and time to progression, and mutational characterisation per patient and per treatment arm. Genetic alterations are categorised per pathway; all included alterations are part of the pathway or directly influencing the pathway. TTP time to progression, BL baseline, OT on treatment, PD progressive disease.

Best response on treatment and time to progression were collected for the total patient population and per treatment arm to correlate genetic alterations and anti-tumour activity (Table 2). The overall response rate was 46%, including one patient with a complete response (CR) and 16 patients with a partial response (PR). The median time to progression (TTP) was 9 months (range 1–26 months). Although significant correlations between mutational status and clinical outcomes were not found in this small sample size, some interesting trends were observed as described below. The genetic alterations per patient and anti-tumour response, including best response (BR) and TTP, is schematically shown in Fig. 1.

**Table 2:** Anti-tumour activity.

	Encorafenib + cetuximab *n* = 17	Encorafenib + cetuximab + binimetinib *n* = 7	Encorafenib + cetuximab + alpelisib *n* = 13	Total *n* = 37
Best response, *n* (%)				
CR	1 (6%)	0 (0%)	0 (0%)	1 (3%)
PR	8 (473%)	3 (43%)	5 (38%)	16 (43%)
SD	6 (35%)	3 (43%)	7 (54%)	16 (43%)
PD	2 (12%)	1 (14%)	1 (8%)	4 (10%)
TTP, median (range), months	8 (1–26)	14 (3–26)	7 (2–16)	9 (1–26)

*CR* complete response, *PR* partial response, *SD* stable disease, *PD* progressive disease, *TTP* time to progression, *n* number of patients.

Paired biopsies were available for 19 patients, of which 16 double paired biopsies and three triple paired biopsies. However, three patients were excluded from the analyses of paired biopsies, because of the lack of overlap in genes tested in the different sequencing methods per time point. In summary, baseline assessment was conducted in 16 non-responding and 15 responding patients and paired analyses comparing different time points in 16 patients (see Fig. S1 for the CONSORT diagram).

### Biomarkers that predict non-response

**Fig. 2:**
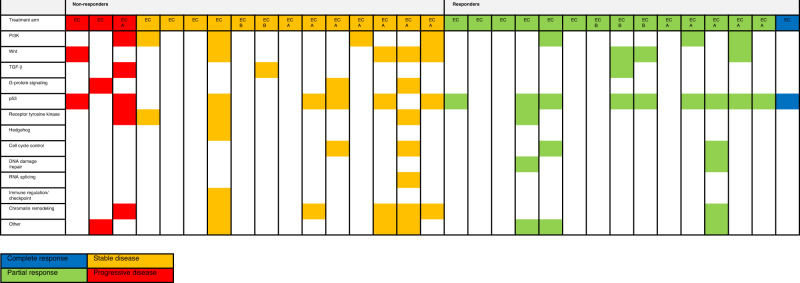
Genetic alteration per pathway in tumours of non-responding patients versus responding patients. Genetic alterations are categorised per pathway; all included alterations are part of the pathway or directly influencing the pathway. EC encorafenib and cetuximab, ECB encorafenib, cetuximab and binimetinib, ECA encorafenib, cetuximab and alpelisib.

The baseline samples of 16 patients with stable (*n* = 13) or progressive disease (*n* = 3) were analysed with the sequencing panels indicated in Table S1 to understand the genetic alterations that predict non-response. These 16 patients were compared to the baseline samples of 15 patients with response, including 14 patients with a PR and one patient with a CR. See Fig. 2 for the specific mutations in responding versus non-responding patients. No mutations other than *BRAF*^*V600E*^ were found in eight patients, four non-responders and four responders. Six mutations were observed in the phosphoinositide-3-kinase (PI3K) pathway among four different non-responders and four mutations were observed among four different responders. Interestingly, *PIK3CA* mutations were found in non-responding patients and *PTEN* mutations in responding patients. Genetic alterations in or directly influencing the WNT pathway were detected in the tumour of five non-responding patients and three responding patients. The WNT pathway mutations in the three responding patients only included the commonly mutated *APC* gene. Given the fact that *APC* (and also *TP53*) mutation is frequent in colon cancer (seen in 35–70% of patients), it is unlikely that APC itself is a biomarker of response to BRAF inhibitor combinations. This means that especially relevant mutations in the WNT pathway or directly influencing the WNT pathway were observed in non-responding patients in the context of intrinsic resistance. Genetic alterations in genes related to chromatin remodelling were found in six non-responding patients and in only one responding patient. Moreover, in three patients mutations were observed in the epidermal growth factor receptor (*EGFR*) or another component of this receptor family (*ERBB2* or *ERBB4*).

Interestingly, if not counting *TP53* and *APC* mutations, 73% of the tumours of non-responding patients were mutated in other genes than *BRAF* or *TP53* and for responding patients this was only 46%. Furthermore, two pathways were mutated in more than one non-responding patients and not in responding patients, including mutations involved in RNA splicing (*n* = 2) and G-protein signalling (*n* = 3). No *KRAS* mutations were found at baseline in these 31 patients, which is not surprising since *BRAF* and *KRAS* mutations are considered mutually exclusive.^22^ In addition, no difference in outcome could be identified between microsatellite stable and instable tumours.

To summarise, genetic alterations in the following genes seem to predict non-response; alterations in or directly influencing the WNT pathway, alterations directly influencing chromatin remodelling, alterations in *EGFR*, components of EGFR or other receptor tyrosine kinases or alterations in *PIK3CA*. These mutations possibly bypass the MAPK pathway in *BRAF*^*V600E*^ mutant CRC. A greater part of non-responding patients had at least one genetic alteration in other genes than *TP53*, *APC* or *BRAF*. Finally, genetic alterations in genes involved in G-protein signalling, immune regulation, RNA splicing and the Hedgehog pathway were only detected in the tumours of non-responding patients, but the significance of this remains uncertain.

### Development of secondary mutations causing resistance

**Fig. 3:**
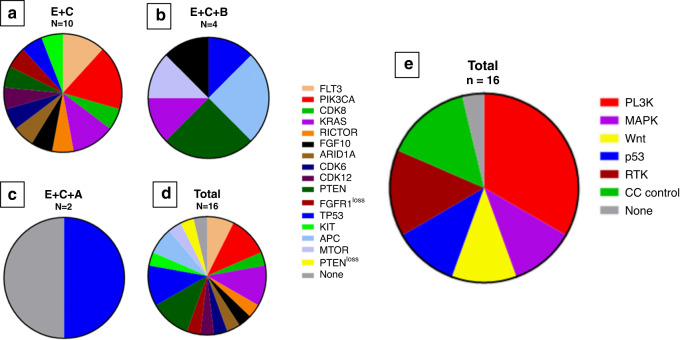
Pie charts of acquired mutations during treatment with MAPK inhibitors, per treatment arm. **a**–**d** Show pie charts with the specific mutations. **e** Shows a pie chart with the involved pathways in which mutations were observed. E encorafenib, C cetuximab, B binimetinib, A alpelisib, N number of patients.

Since acquired resistance remains a major problem in the treatment with MAPK inhibitors, we explored the development of secondary mutations causing resistance by looking into available paired biopsies of 16 patients. From these 16 patients, ten were treated with encorafenib and cetuximab, four with encorafenib, cetuximab and binimetinib and two with the combination of encorafenib, cetuximab and alpelisib. Only mutations were included which were at least tested in molecular assessments at two time points. Several interesting trends were observed during the development of secondary resistance mutations (Fig. 3). One out of 16 patients did not develop a de novo mutation in a gene tested before and after treatment. The other 15 patients developed genetic alterations in different pathways. Four mutations were observed in receptor tyrosine kinases or their ligands (FGF, ERBB), which could lead to resistance by activation of upstream signalling. Six out of 15 patients (40%) developed mutations in the PI3K pathway, of which one patient was treated with the triple combination including alpelisib. This patient simultaneously developed genetic alterations in the MAPK and WNT pathways. We cannot exclude that the biopsy contained two clones that had acquired independent resistance mutations.

### Heterogeneity

Besides interpatient variability in secondary mutations, heterogeneity was also observed within individual patients. For one patient treated with the triple combination of encorafenib, cetuximab and binimetinib, tumour tissue from three different liver metastases was available on treatment. All metastases harboured *BRAF*^*V600E*^, *APC*^R1450*^ and *PIK3CA*^*E545K*^ mutations, but only two of the lesions harboured a *PTEN* mutation and one of these lesions a *KRAS*^*G12V*^ mutation. Interestingly, the *PTEN* mutations were not identical, including a *PTEN*^*R173C*^ and *PTEN*^R233*^ mutation, marking the heterogeneity of the disease. When correlating these genetic alterations with radiological assessment, the lesion with the *PTEN*^R233*^ and *KRAS*^*G12V*^ mutation was progressive while the other two liver metastasis were in regression. The best response for this patient was stable disease with a TTP of 22 months. This patient demonstrates the heterogeneous character of the disease and heterogeneous response to treatment of different metastatic lesions.

In summary, the development of acquired resistance is common with intra- and interpatient heterogeneity and secondary mutations are observed on different levels in the MAPK pathway and interconnected pathways.

Taken all results together, genetic alterations in *EGFR* and in *PIK3CA* are associated with non-response. A greater fraction of non-responders (75%) versus responders (46%) had at least one genetic alteration in other genes than *TP53*, *APC* or *BRAF*. Secondary resistance mutations (*n* = 16 patients) were observed most frequently in the PI3K pathway (*n* = 6) and in receptor tyrosine kinases (*n* = 4), leading to increased upstream signalling.

## Discussion

In this retrospective cohort study, patterns of intrinsic and acquired resistance in patients with *BRAF*^*V600E*^ mutated metastatic CRC treated with double or triple combinations of inhibitors of the MAPK and PI3K pathways were observed. The majority of mutated pathways at baseline in our cohort study were similar to findings described in the literature. A total of six prior published clinical trials report molecular results of tumour tissue and circulating free DNA (cfDNA) in patients with *BRAF*^*V600E*^ mutated metastatic CRC receiving therapies consisting of a backbone of BRAF and EGFR inhibition or BRAF inhibition monotherapy.^7,10,13,18,23,24^ No difference in outcome was described between microsatellite stable and instable tumours in this cohort nor in literature.^7,12,13^ The genetic alterations identified for acquired resistance arose in genes directly or indirectly activating signalling via the MAPK pathway or cross-linked pathways. In our cohort study, genetic alterations in or directly influencing the WNT pathway, directly influencing chromatin remodelling, in the *PI3K* pathway, and upstream in EGFR or other receptor tyrosine kinases seem to predict for non-response as these mutations probably are considered driver mutations in *BRAF*^*V600E*^ mutant CRC. The earlier investigation of 15 molecularly analysed *BRAF*^*V600E*^ CRC tumour samples of patients treated with the BRAF inhibitor dabrafenib and MEK inhibitor trametinib harboured alterations in the WNT and p53 pathways without a clear correlation with treatment outcome. Five out of 15 tumours had mutations in *PIK3CA* of which 60% of patients had a PR or CR. However, no clear correlation was reported for *PTEN* loss or EGFR expression and progression free survival.^13^ Remarkably, in our study cohort and in cfDNA profiling by Hong *et al*. it was observed that resistance might be caused by mutations in the PI3K or genes influencing the WNT pathway or at the level of EGFR signalling, resulting in signalling through these pathways.^23^ Activation of the WNT pathway or upstream receptor tyrosine kinases plays a pivotal role in epithelial-to-mesenchymal transition (EMT), often associated with a CSM4 subtype, in colorectal cancers leading to an adverse prognostic phenotype causing resistance to anti-cancer therapies. Another pathway involved in the development of the EMT subtype is TGF-β, which was probably activated in a minority of patients due to mutations in the *SMAD* gene.^25^ Surprisingly, Middleton *et al*. has shown that the majority of CSM4 subtype *BRAF*^*V600E*^ mutant CRC represents a BM1 signature with a better response to combined treatment with dabrafenib, trametinib and panitumumab.^18^ Unfortunately, it was not feasible to actually determine molecular subtypes BM1 and BM2 in our patient cohort due to restrictions in sequencing data. It would nevertheless be very interesting to define those subtypes in future research on BRAF/MEK and EGFR inhibition to confirm if screening for BM1 or BM2 at baseline could be used as predictive biomarker for sensitivity to targeted treatments in *BRAF*^*V600E*^ mutant CRC.

A total of three out of 16 patients in this cohort developed *KRAS* mutations on disease progression. Focal amplification of* KRAS* was earlier reported in one post-progression biopsy of a patient treated with RAF/MEK inhibition and in cfDNA samples of patients treated with vemurafenib and panitumumab.^12,26^ In addition, *KRAS* or *NRAS* clones or subclonal *RAS* mutations were detected in respectively 48% and 21% of patients on the time of disease progression.^24^ *KRAS* mutations activate CRAF leading to sustained phosphorylation of ERK and resistance, despite combined BRAF and EGFR or MEK inhibition.^26^ The simultaneous presence of *KRAS* and *BRAF* mutations also implies disease heterogeneity, since *KRAS* and *BRAF* mutations are mutually exclusive during primary tumour development.^22^ Clones sensitive and resistant to treatment might be present at the same time, which must be taken into account by switching to the next line of anti-tumour therapy. Adding an inhibitor to the current treatment regimen after development of resistance is recommendable to enhance treatment duration, if toxicity of the new drug combination is expected to be manageable. In the case of disease heterogeneity and expected severe or unpredictable toxicity of a combination, it might be better to start an alternating treatment regimen. This approach is currently investigated in resistant *BRAF*^*V600E*^ mutated melanoma. In a proof of concept study, patients are treated with the histone deacetylase inhibitor vorinostat for 14 days upon resistance and thereafter BRAF and/or MEK inhibitors are reintroduced.^27^ This cohort contains unique data on paired molecular analyses in tumour tissue for patients with *BRAF*^*V600E*^ mutant metastatic CRC treated with encorafenib and cetuximab with or without binimetinib or alpelisib. Since this treatment combination was recently approved by the FDA and the European Medicines Agency (EMA), the data are considered highly relevant for clinical practice. Despite these unique data, our study has some limitations. No statistically significant differences between non-responders and responders could be found due to the combination of the small sample size, difference in sequencing methods and the lack of molecular analyses on three different time points for all patients. Since molecular analysis on the different time points was not always performed in tumour tissue of the same lesion, it might be that mutations detected were not necessarily newly developed. Due to tumour heterogeneity, these mutations could already have been present at start of treatment. To strengthen our data, we decided to perform the analyses on paired biopsies in 16 patients to improve robustness of the results. Despite the presence of uncertainties, the trends in our and earlier published data provide insight into the mechanisms of resistance in this specific patient group and might generate opportunities for future studies.

In conclusion, our findings show that genetic alterations causing intrinsic and acquired resistance in this patient cohort were observed before and upon treatment with BRAFV600E targeted therapies. The genetic alterations revealed for intrinsic and acquired mutations arose in genes directly or indirectly activating signalling via the MAPK pathway or cross-linked pathways. Intrinsic and acquired resistance mechanisms are heterogeneous with a high intra- and interpatient variability. Based on these results, we suggest comprehensive molecular screening of *BRAF*^*V600E*^ mutant metastatic CRC before start of first-line treatment in the palliative setting. Furthermore, it might be considered to closely monitor genetic alterations and accordingly switching therapy to a combined simultaneous or alternating treatment with a backbone of BRAF and EGFR inhibition combined with an inhibitor of the genetic alterations to optimise duration of treatment.

Monitoring of genetic alterations and switching therapies accordingly of course, could not be considered part of standard therapy but should be the scope of future studies. The transcriptional context, identification of responding and non-responding subtypes such as BM1 and BM2, real-time monitoring of tumour DNA and the effect of accordingly changing treatment strategies on response should be part of that.

## Supplementary information

supplementary tables en figure

## Data Availability

The authors are committed to share sequencing data and patient characteristics in an anonymised manner according to applicable privacy regulations and laws. All data requests are reviewed and approved by the Institutional Review Board of the NKI-AVL on the basis of scientific relevance.
