# Supplementary material for: Mutational profiles associated with resistance in patients with BRAFV600E mutant colorectal cancer treated with cetuximab and encorafenib +/− binimetinib or alpelisib
Source: Br J Cancer. 2020 Nov 18;124(1):176–82. doi: 10.1038/s41416-020-01147-2 (PMC7782586; doi:10.1038/s41416-020-01147-2)
Supplement: Supplementary file 1 — supplementary tables en figure [file 41416_2020_1147_MOESM1_ESM.pdf]

Supplementary table S1. Genes included in the different sequencing methods.

Sequencing panel	Tested genes
AVL panel v1.1<br><br>(Antoni van Leeuwenhoek, Amsterdam, the Netherlands)	Amino acid positions of AKT1 (p.E17), BRAF (p.G466, p.G469, p.L597, p.V600, p.K601), DDR2 (p.S768), EGFR (p.G719, p.G721, p.V774, p.R776, p.T790, p.G796, p.A840, p.V843, p.L858, p.A859, p.K860, p.L861, p.G863, p.H870, p.A871), MEK1 (p.Q56, p.K57, p.D67), PIK3CA (p.E542, p.E545, p.Q546, p.H1047), KRAS (p.A11, p.G12, p.G13, p.V14, p.Q61, p.K117, p.A146), NRAS (p.G12, p.G13, p.A59, p.Q61, p.R68, p.K117, p.A146)
AVL panel v1.2<br><br>(Antoni van Leeuwenhoek, Amsterdam, the Netherlands)	Amino acid positions of AKT1 (p.E17), BRAF (p.G466, p.G469, p.L597, p.V600, p.K601), DDR2 (p.S768), EGFR (p.G719, p.G721, p.V774, p.R776, p.T790, p.G796, p.A840, p.V843, p.L858, p.A859, p.K860, p.L861, p.G863, p.H870, p.A871), MEK1 (p.Q56, p.K57, p.D67), PIK3CA (p.E542, p.E545, p.Q546, p.Q1042, p.H1047, p.T1052) KRAS(p.A11, p.G12, p.G13, p.V14, p.A59, p.Q61, p.K117, p.A146), NRAS (p.G12, p.G13, p.A59, p.Q61, p.R68, p.K117, p.A146)
TSACP v1.0<br>(MiSeq; Illumina, San Diego, CA, USA)	Hotspot mutations in ABL1, AKT1, ALK, APC, ATM, BRAF, CDH1, CDKN2A, CSF1R, CTNNB1, EGFR, ERBB2, ERBB4, FBXW7, FGFR1, FGFR2, FGFR3, FLT3, GNA11, GNAQ, GNAS, HNF1A, HRAS, IDH1, JAK2, JAK3, KDR, KIT, KRAS, MET, MLH1, MPL, NOTCH1, NPM1, NRAS, PDGFRA, PIK3CA, PTEN, PTPN11, RB1, RET, SMAD4, SMARCB1, SMO, SRC, STK11, TP53, VHL<br>Gene amplifications in EGFR, ERBB2 (HER2), MET
Oncocarta panel v1.0 (Agene; San Diego, CA, USA)	Relevant regions of genes AKT1, AKT2, BRAF, CDK, EGFR, ERBB2, FGFR1, EGR3, FLT3, HRAS, JAK2, KIT, KRAS, MET, NRAS, PDGFRA, PIK3CA, RET
Foundation Medicine (Cambridge, Massachusetts, USA)	Relevant regions of genes ABL1, ACVR1B, AKT1, AKT2, AKT3, ALOX12B, ALK, AMER1, APC, AR, ARAF, ARFRP1, ARID1A, ASXL1,ATM, ATR, ATRX, AURKA, AURKB, AXIN1, AXL, BAP1, BARD1, BCL2, BCL2L1, BCL2L2, BCL6, BCOR, BCORL1, BRAF, BRCA1, BRCA2, BRD4, BRIP1, BTG1, BTG2, BTK, CALR, CARD11, CASP8, CBFB, CBL, CCND1, CCND2,

	CCNE1, CD22, CD274, CD70, CD79A, CD79B, CDC73, CDH1, CDK4, CDK6, CDK8, CDK12, CDKN1A, CDKN1B, CDKN2A, CDKN2B, CDKN2C, CEBPA, CHEK1, CHEK2, CIC, CREBBP, CRKL, CSF1R, CSF3R, CTCF, CTNNA1, CTNNB1, CCND3, CUL3, CUL4A, CXCR4, CYP17A1, DAXX, DDR1, DDR2, DIS3, DNMT3A, DOT1L, EED, EGFR, EP300, EPHA3, EPHB1, EPHB4, ERBB2, ERBB3, ERBB4, ERCC4, ERG, ERRFI1, ESR1, EZH2, FAM46C, FANCA, FANCC, FANCG, FANCL, FAS, FBXW7, FGF3, FGF4, FGF6, FGF10, FGF12, FGF14, FGF19, FGF23, FGFR1, FGFR2, FGFR3, FGFR4, FH, FLCN, FLT1, FLT3, FOXL2, FUBP1, GABRA6, GATA3, GATA4, GATA6, GID4, GNA11, GNA13, GNAQ, GNAS, GRM3, GSK3B, H3F3A, HDAC1, HGF, HNF1A, HRAS, HSD3B1, ID3, IDH1, IDH2, IGF1R, IKBKE, IKZF1, INPP4B, IRF2, IRF4, IRS2, JAK1, JAK2, JAK3, JUN, KDM5A, KDM5C, KDM6A, KDR, KEAP1, KEL, KIT, KLHL6, KMT2A, KMT2D, KRAS, LTK, LYN, MAF, MAP2K1, MAP2K2, MAP2K4, MAP3K1, MAP3K13, MAPK1, MCL1, MDM2, MDM4, MED12, MEF2B, MEN1, MERTK, MET, MITF, MKNK1, MLH1, MLL, MLL2, MPL, MRE11A, MSH2, MSH3, MSH6, MST1R, MTAP, MTOR, MUTYH, MYC, MYCL, MYCN, MYD88, NBN, NF1, NF2, NFE2L2, NFKBIA, NKX2-1, NOTCH1, NOTCH2, NOTCH3, NPM1, NRAS, NT5C2, NTRK1, NTRK2, NTRK3, P2RY8, PALB2, PARK2, PARP1, PARP2, PARP3, PAX5, PBRM1, PDCD1, PDCD1LG2, PDGFRA, PDGFRB, PDK1, PIK3C2B, PIK3C2G, PIK3CA, PIK3CB, PIK3R1, PIM1, PMS2, POLD1, POLE, PPARG, PPP2R1A, PPP2R2A, PRDM1, PRKAR1A, PRKCI, PTCH1, PTEN, PTPN11, PTPRO, QKI, RAC1, RAD21, RAD51, RAD51B, RAD51C, RAD51D, RAD52, RAD54L, RAF1, RARA, RB1, RBM10, REL, RET, RICTOR, RNF43, ROS1, RPTOR, SDHA, SDHB, SDHC, SDHD, SETD2, SF3B1, SGK1, SMAD2, SMAD4, SMARCA4, SMARCB1, SMO, SNCAIP, SOCS1, SOX2, SOX9, SPEN, SPOP, SRC, STAG2, STAT3, STK11, SUFU, SYK, TEK, TBX3, TET2, TGFBR2, TIPARP, TNFAIP3, TNFRSF14, TP53, TSC1, TSC2, TYRO3, U2AF1, VEGFA, VHL, WHSC1, WHSC1L1, WT1, XRCC2, XPO1, ZNF217, ZNF703
Trusight tumor 15 kit<br>(Illumina, San Diego, CA, USA)	Hotspot mutations in AKT exon 3, BRAF exon 15, EGFR exon 12,18,19,20,21, HER2 (ERBB2) exon 17-21, 24, 26, FOXL2-gen exon 1, GNA11 exon 5, GNAQ exon 5, KIT exon

	8-11, 13, 14, 17, 18, KRAS exon 2-4, MET exon 16, 18 , 20 NRAS exon 1-4, PDGFRA exon 12, 14, 18, PIK3CA 9 en 20, RET exon 16, TP53 exon 1-11
Ampliseq Cancer hotspot panel v2- SOC v1<br><br>(Illumina, San Diego, CA, USA) (Standard of Care genes added by Antoni van Leeuwenhoek, Amsterdam, the Netherlands)	Relevant regions of genes BL1, AKT1, ALK,APC, ATM, BRAF, CDH1, CDKN2A, CSF1R, CTNNB1, EGFR, ERBB2, ERBB4, EZH2, FBXW7, FGFR1, FGFR2, FGFR3, FLT3, GNA11, GNAQ, GNAS, HNF1A, HRAS, IDH1, IDH2, JAK2, JAK3, KDR, KIT, KRAS, MET, MLH1, MPL, NOTCH1, NPM1, NRAS, PDGFRA, PIK3CA, POLE, PTEN, PTPN11, RB1, RET, ROS1, SMAD4, SMARCB1, SMO, SRC, STK11, TP53, VHL
Ampliseq Cancer hotspot panel v3<br><br>(Thermo Fisher, Watham, MA, USA)	ARAF exon 7, 10, CTNNB1 exon 1, 2, 4, 7, 8, 12, 15, KRAS exon 2-4, HRAS exon 2, 3, NRAS exon 2-4, BRAF exon 11, 15, EGFR exon 2, 7, 15, 18-21, GNAQ exon 5, GNAS exon 8, 9, H3F3A exon 2, H3F3B exon 2, IDH1 exon 4, IDH2 exon 4, KIT exon 2, 9-18, YD88 exon 3b, 5, MUTYH exon 7,13, PDGFRA exon 12, 14, 15, 18, 23, PIK3CA exon 2, 5, 6-10, 14, 18 21, POLE exon 9, 11, 13, 14, RET exon 10-12, 15, 16, TP53 exon 1-11<br>Hotspot mutations in ABL1, AKT1, ALK, APC, ATM, CARD11, CD79A, CD79B, CDH1, CDKN2A, CSF1R, CTNBB1, ERBB2, ERBB4, EZH2, FBXW7, FGFR1, FGFR2, FGFR3, FLT3, GNA11, HNF1A, JAK2, JAK3, KDR, MET, MLH1, MPL, NOTCH1, NPM1, PTEN, PTPN11, RB1, SMAD4, SMARCB1, SMO, SRC, STK11, VHL
NGS Path v2D<br>(Radboud UMC, Nijmegen, the Netherlands)	Relevant regions of genes AKT1, AKT2, AKT3, ALK, ARAF, BRAF, DDR2, EGFR, ERBB2, GNAS, GNAQ, GNA11, HRAS, IDH1, IDH2, JAK2, KIT, KRAS, MAP2K1, MET, MTOR, NRAS, PDGFRA, PIK3CA, POLE, PTEN, RAF1, ROS1, TP53

Supplementary table S2. Case-series of BRAFV600E mutant colorectal cancer patients treated with double or triple targeted therapy inhibiting the MAPK pathway in the BEACON CRC phase III study (Kopetz et al. NEJM 2019) and genetic alterations at baseline, newly developed genetic alterations during treatment and at time of progression.

Abbreviations: yrs, years; MSI microsatellite instable; MSS, microsatellite stable; M, male; F, Female; SD, stable disease; BL, baseline; OT, on treatment; PD, progressive disease; -, no other genetic alterations than BRAFV600E; NA, no data available.

Mutations in bold were analyzed at more than one time point.

‖These mutations were found in another lesion, which was progressive at time of biopsy, than the other reported mutations.

Patient	Age (yrs)	Sex	Prior treatment lines	MSI status	Best response	Time on treatment (months)	Genetic alterations at baseline	Genetic alterations on treatment	Genetic alterations at progressive disease	Analytical method
Double combination: encorafenib+cetuximab										
9	68	F	2	MSS	SD	5	-	**TP53^{G293fs}**	**TP53^{G293fs}**	BL: AVL panel v1.2, TSACP v 1.0<br>OT and PD: Ampliseq Cancer hotspot panel v2- SOC v1
13	51	F	3	MSS	PR	1	AKT1^{splice}<br>ERBB2^{A689L}<br>ERBB2^{I655V}<br>KIT^{M541L}	NA	NA	BL: Trusight tumor 15 kit<br>OT and PD: NA
16	59	F	2	MSS	CR	15	**TP53^{H179R}**	**TP53^{H179R}**<br>**PIK3CA^{E542K}**	**TP53 ^{H179R}**<br>**PIK3CA^{E542K}**<br>RNF43^{supl}	BL: TSACP v1.0<br>OT: Ampliseq Cancer hotspot panel v2- SOC v1

									KRAS^{ampl}<br>EZH2^{del}<br>CHECK2^{loss}	PD: Whole genome sequencing
17	55	F	2	MSS	PR	4	**TP53**$^{\mathbf{splice}}$	**TP53**$^{\mathbf{splice}}$<br>KIT^{R634W}	NA	BL: at least hotspot mutations in TP53, KRAS and BRAF<br>OT: Ampliseq Cancer hotspot panel v2- SOC v1<br>PD: NA
18	66	F	2	MSS	PD	2	APC^{S1411Afs*4}<br>TP53^{T234A}	NA	NA	BL: AVL panel v1.1 and TSACP v1.0<br>OT and PD: NA
20	66	F	3	MSI	SD	11	-	EP300^{P925T}<br>KDM5A^{R1157H}<br>**PIK3CA**$^{\mathbf{R108H}}$<br>SF3B1^{R831Q}<br>APC^{Q2315*}<br>CREBBP^{splice}<br>BRCA2^{F2560fs*5}<br>CTNNA1^{splice}<br>CTNNA1^{Y843*}	NA	BL: Oncocarta panel v1.0<br>OT: Foundation Medicine<br>PD: NA

								SMARCA4^{R397*}<br>SMARCA4^{G1478*}<br>**FLT3$^{P986fs*9+}$**<br>GATA3$^{S437fs*9+}$<br>MLH1^{Y157fs*3}<br>PTCH1^{S1203fs852}<br>TSC2$^{P1042fs+11}$		
21	65	F	2	NA	PR	14	-	**PTEN^{loss}**<br>**RNF43^{I48T}**<br>**TP53^{E366fs*9}**<br>**SMAD4^{dupl}**<br>FGF14^{ampl}<br>IRS2^{ampl}<br>CDK8^{ampl}<br>FLT3^{ampl}	**PTEN^{loss}**<br>**RNF43^{I48T}**<br>**TP53^{E366fs*9}**<br>**SMAD4^{dupl}**	BL: Oncocarta panel v1.0<br>OT and PD: Foundation Medicine
22	61	M	1	MSS	PR	3	NA	**TP53^{R273H}**<br>**Notch4$^{Y1359fs*10+}$**<br>**MYC^{ampl}**<br>**PAX5^{V26G}**	**TP53^{R273H}**<br>**Notch4^{Q1360fs*102}**<br>**MYC^{ampl}**<br>RICTOR^{ampl}	BL: NA<br>OT and PD: Foundation Medicine

								**PRKDC^{fusion}**	FGF10^{ampl}<br>ARID1A^{trunc}<br>**KRAS^{G12R}**	
23	63	M	2	NA	PR	26	NA	**FBXW7^{R465C}**<br>**TP53^{M169fs*5}**<br>**MCL1^{ampl}**<br>**RICTOR^{ampl}**<br>**FGF10^{ampl}**<br>**PIK3CA^{H1047R}**<br>**HGF^{ampl}**<br>**CDK6^{ampl}**	**FBXW7^{R465C}**<br>**TP53^{M169fs*5}**<br>**MCL1^{ampl}**<br>**RICTOR^{ampl}**<br>**FGF10^{ampl}**	BL: NA<br>OT and PD: Foundation Medicine
24	63	F	2	NA	SD	4	-	FGF3<br>CREBBP^{Q1152*}<br>NF1^{A2617V}<br>BCORL1^{Y814*}<br>BCORL1^{Q888fs*29}<br>MLL2^{splice}<br>MLL2^{N2977fs*13}<br>RAD50^{R1200*}	NA	BL: Oncocarta panel v1.0<br>OT: Foundation MedicinePD: NA

								WT1^{Y2618}<br>**CDK12$^{G1461fs*31+}$**<br>CIC^{P1116fs*45}<br>MEN1^{R521fs*43}<br>PBRM1^{F993fs*15}<br>BCOR^{S336fs*45}<br>BCORFL1^{Y814*}<br>BCORFL1^{Q888fs*29}<br>BRIP1^{N1087fs*4}<br>FBXW7^{R473fs*4}		
25	64	M	2	NA	PR	13	-	**EGFR^{V292L}**<br>**TP53^{R213*}**	**EGFR^{V292L}**<br>**TP53^{R213*}**<br>PTEN^{loss}	BL: at least KRAS and BRAF<br>OT: Foundation Medicine<br>PD: TSACP v1.0
26	73	M	1	MSS	PR	10	**TP53^{R175H}**<br>MUTYH^{G382D}<br>VEGFA^{ampl}<br>FGFR1^{ampl}	NA	**TP53^{R175H}**	BL: Foundation Medicine<br>OT: NA<br>PD: TSACP v1.0, NRAS codon 117 and 146
29	62	M	3	NA	SD	15	NA	APC^{T1556fs*3}<br>APC^{L620fs*13}	NA	BL and PD: NA<br>OT: Foundation Medicine

								FBXW7^{R367*}<br>PIK3R1^{K459del}<br><br>ASXL1^{G645fs*58}<br>KDM5C^{G1504fs*40}<br>RB1^{M484fs*8}		
33	64	F	1	NA	PR	12	TP53^{R213*}<br>IRS^{ampl}<br>CDK8^{ampl}<br>SPEN^{T448fs*25}<br>SPEN^{G1860fs*9}	NA	NA	BL: Foundation Medicine<br>OT and PD: NA
36	57	M	1	NA	PR	4	-	APC^{E1554*}<br>APC^{C661fs*12}<br>EPHA5^{G287R}<br>TP53^{G245S}<br>BCORL1^{T63fs*53}	NA	BL: Oncocarta panel v1.0<br>OT: Foundation Medicine<br>PD: NA
43	60	F	1	NA	SD	3	CD79A^{R131fs*61}<br>CTCF^{A175T}<br>EGFR^{R776C}	NA	NA	BL: Foundation Medicine<br>OT and PD: NA

							$EPHB4^{A800T}$<br>$MLL3^{K2797fs*26}$<br>$PIK3CA^{P104L}$<br>$PTCH1^{G43E}$<br>$TP53^{R273C}$<br>$TP53^{splice}$<br>$ARID1A^{G276fs*87}$<br>$BAP1^{W5*}$<br>$FAM123B^{E112*}$<br>$MLL2^{R2235fs*29}$<br>$SPEN^{T448fs*25}$<br>$SPEN^{G1860fs*9}$<br>$TSC2^{splice}$			
46	73	F	2	NA	SD	6	-	NA	NA	BL: AVL panel v1.1<br>OT and PD: NA
50	52	F	1	NA	PD	1	$GNAS^{R201H}$<br>$CDH1^{R598*}$	NA	NA	BL: Foundation Medicine<br>OT and PD: NA
51	54	F	3	MSS	SD	8	-	NA	NA	BL: NRAS exon 2-4, BRAF exon 11, 15, KRAS exon 2-4

										OT and PD: NA
52	68	F	3	NA	PR	17	-	NA	NA	BL: NRAS exon 2-4, BRAF exon 11, 15, KRAS exon 2-4<br>OT and PD: NA
53	57	F	3	MSS	PD	3	-	NA	NA	BL: NRAS exon 2-4, BRAF exon 11, 15, KRAS exon 2-4<br>OT and PD: NA
Triple combination: encorafenib+cetuximab+binimetinib										
1	69	M	3	MSS	SD	26	NA	**TP53**^{S1465fs}<br>**APC**^{G245D}	**TP53**^{S1465fs}<br>**APC**^{G245D}	BL: NA<br>OT: TSACP v1.0<br>PD: TSACP v1.0
2	47	F	3	MSS	SD	22	**APC**^{R1450*}<br>**PIK3CA**^{E545K}	**APC**^{R1450*}<br>**PIK3CA**^{E545K}<br>PTEN^{R173C}<br>‖KRAS^{G12V}<br>‖PTEN^{R233*}	NA	BL: NRAS exon 4, codon 117 and 146, TSACP v1.0<br>OT: AVL panel v1.2, TSACP v1.0<br>PD: NA
3	45	M	2	MMS	PR	26	**TP53**^{R175H}	**TP53**^{R175H}<br>APC^{C1502*}<br>KRAS^{G12A}<br>MET^{ampl}	NA	BL: TP53 exon 2-10, TSACP v1.0<br>OT: TSACP v1.0, NRAS exon 4, codon 117 and 146<br>PD: NA

4	46	F	2	MSS	PR	4	$TP53^{P151S}$<br>$APC^{T1556fs}$<br>$SMAD4^{G365D}$	NA	NA	BL: TSACP v1.0, NRAS exon 4 codon 117 and 146<br>OT and PD: NA
5	68	F	3	MSS	PR	8	-	NA	NA	BL: AVL panel v1.2, TSACP v`.0, NRAS exon 4, codon 117 and 146<br>OT and PD: NA
6	56	F	3	MSS	SD	7	-	NA	NA	BL: AVL panel v1.2<br>OT and PD: NA
7	60	M	2	MSS	SD	9	-	NA	NA	BL: AVL panel v1.2<br>OT and PD: NA
8	65	F	2	NA	SD	10	-	NA	NA	BL: KRAS exon 2-4, BRAF exon 11, 15, NRAS exon 2-4<br>OT and PD: NA
10	38	M	2	MSS	SD	12	$SMAD4^{A361H}$	NA	NA	BL: Ampliseq Cancer hotspot panel v3- SOC v1<br>OT and PD: NA
11	67	F	2	MSS	PR	7	-	NA	NA	BL: KRAS exon 2-4, BRAF exon 11, 15, NRAS exon 2-4
12	43	F	2	MSS	PD	3	-	$TP53^{D259Y}$<br>$SMAD4^{D355V}$	NA	BL and OT: Ampliseq Cancer hotspot panel v2- SOC v1

										PD: NA
14	62	F	3	MSS	SD	8	-	-	NA	BL and OT: AVL panel v1.2<br>PD: NA
15	74	M	2	MSS	PR	7	**TP53^{R282W}**<br>APC^{R1450*}	NA	**TP53^{R282W}**<br>MTOR^{I1973F}	BL: TSACP v1.0<br>OT: NA<br>PD: NGS Path v2D
Triple combination: encorafenib + cetuximab + alpelisib										
19	60	F	1	MSS	SD	3	-	NA	**TP53^{G245S}**<br>APC^{E1554*}<br>APC^{C661fs*12}<br>EPHA5^{G287R}<br>BCORL1^{T63fs*53}	BL: Colon AVL panel<br>OT: NA<br>PD: Foundation Medicine
27	75	M	2	NA	SD	31	-	-	-	BL, OT and PD: Colon AVL panel
28	70	F	2	NA	PR	5	-	NA	NA	BL: Oncocarta panel v1.1<br>OT and PD: NA
30	64	M	2	NA	SD	4	TP53^{R306*}<br>MYC^{ampl}<br>MYST3^{ampl}	NA	NA	BL: Foundation Medicine<br>OT and PD: NA
31	46	M	1	NA	PR	4	-	NA	APC^{T1556fs*3}	BL: Colon AVL panel

									**KRAS**$^{\mathbf{G12V}}$<br>STAT4^{R320Q}<br>TP53^{R213*}<br>PBRM1^{S295fs*5}<br>PTEN^{loss}<br>SMAD2^{loss}	OT: NA<br>PD: Foundation Medicine
32	59	F	3	NA	SD	3	-	NA	NA	BL: Oncocarta panel v1.0 and TSACP v1.0<br>OT and PD: NA
34	69	F	2	MSS	SD	17	TP53^{K132N}<br>GNAS^{R201H}<br>CCND3^{ampl}	NA	-	BL: Foundation Medicine<br>OT: NA<br>PD: AVL panel v1.2
35	63	F	2	NA	PR	5	TP53^{R282W}<br>PTEN^{R130*}	NA	NA	BL: Foundation Medicine<br>OT and PD: NA
37	65	F	1	MSS	SD	14	**PIK3CA**$^{\mathbf{E542K}}$	NA	**PIK3CA**$^{\mathbf{E542K}}$<br>TP53^{R282W}<br>SOX9^{Q375*}<br>APC^{H791fs*7}<br>APC^{S1505fs*1}	BL: AVL panel<br>OT: NA<br>PD: Foundation medicine

38	63	M	2	MSI	SD	3	-	NA	NA	BL: Colon AVL panel<br>OT and PD: NA
39	69	F	2	MSI	SD	5	-	NA	NA	BL: KRAS exon 1, BRAF exon 15, EGFR exon 19, 21, PIK3CA exon 9, 20<br>OT and PD: NA
40	66	F	1	MSS	SD	10	-	NA	NA	BL: Oncocarta panel v1.0<br>OT and PD: NA
41	47	M	1	MSS	PR	10	APC^{Q978*}<br>$PBRM1^{R298*}$<br>$TP53^{L257R}$<br>$FBXW7^{E327*}$	NA	NA	BL: Foundation Medicine<br>OT and PD: NA
42	68	F	1	NA	PD	2	$TP53^{R306*}$<br>$PIK3CA^{Q546P}$<br>$ERBB2^{ampl}$<br>$TOP2A^{ampl}$<br>$SMAD4^{loss}$	NA	NA	BL: Foundation Medicine<br>OT and PD: NA
44	59	M	1	NA	SD	2	$BLM^{N515fs*16}$<br>$CCNE1^{A410V}$<br>$CDH1^{P126fs*89}$	NA	NA	BL: Foundation Medicine<br>OT and PD: NA

							ERBB4^{D861V}<br>GNAS^{R201C}<br>HNF1A^{P291fs*51}<br>MLL3^{K2797fs*26}<br>ARID1A^{P225fs*175}<br>CREBBP^{P1946fs*30}<br>FLCN^{splice}<br>LRP1B^{K3773fs*26}<br>MLL2^{A1390fs*27}<br>QKI^{K134fs*14}<br>RNF43^{G659fs*41}<br>SLIT2^{T415fs*21}			
45	38	F	2	MSS	SD	6	PIK3CA^{E542K}<br>TP53^{C176F}<br>MYC^{ampl}<br>RNF43^{D96fs*62}	NA	NA	BL: Foundation Medicine<br>OT and PD: NA
47	54	F	1	MSS	PR	5	CDH2^{V491I}<br>FANCA^{A586T}<br>FLCN^{A324V}	NA	NA	BL: Foundation Medicine, AVL panel v1.1<br>OT and PD: NA

							$TP53^{splice}$<br>$TP53^{D228fs*1}$<br>$MYST3^{ampl}$<br>$CDKN2A^{loss}$<br>$CDKN2B^{loss}$			
48	60	F	1	MSS	PR	5	$PIK3CA^{H1047R}$<br>APC^{E1353*}<br>APC^{E1554*}<br>$PTEN^{Y188*}$<br>$TP53^{I195T}$	NA	NA	BL: TSACP v1.0<br>OT and PD: NA
49	67	F	1	MSS	PR	16	$TP53^{R175H}$	NA	NA	BL: TSACP v1.0<br>OT and PD: NA

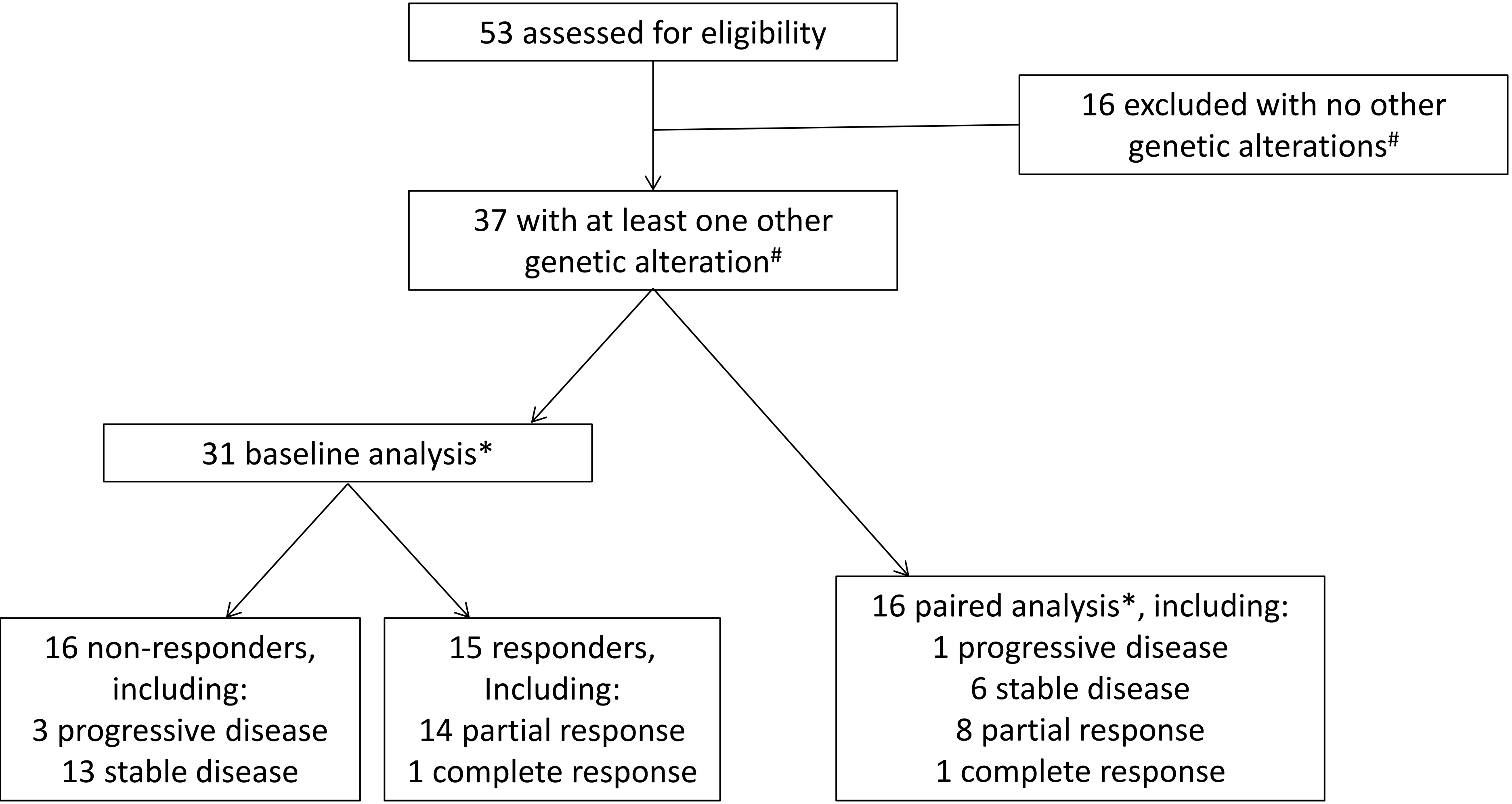

Figure S1. CONSORT diagram for this retrospective cohort study.

# Other genetic alterations at one time point, besides the known BRAF^{V600E} mutation.

** Patients might be included in the baseline and paired analyses comparing different time points.*
